# Retrospective Evaluation of Pediatric Emergency Department Visits of Children With Medical Complexity in a Tertiary Care Center in Italy

**DOI:** 10.1097/PEC.0000000000003463

**Published:** 2025-10-01

**Authors:** Anna Zanin, Chiara La Piana, Gloria Brigiari, Dario Gregori, Antuan Divisic, Silvia Bressan, Tiziana Zangardi, Susanna Masiero, Franca Benini

**Affiliations:** * Palliative Care and Pain Service, Department of Women’s and Children’s Health; † Department of Women’s and Children’s Health; ‡ Unit of Biostatistics, Epidemiology and Public Health, Department of Cardiac, Vascular Sciences and Public Health; § Pediatric Emergency Department, Department of Women’s and Children’s Health, University of Padua, Padua, Italy

**Keywords:** children with medical complexity, children with complex chronic conditions, pediatric palliative care

## Abstract

**Objective:**

Children with medical complexity are a growing population with multiple conditions, medical device dependency and frequent need for emergency department (ED) visits; their care and management in an emergency setting may be challenging. The pediatric palliative care (PPC) network aims to address the needs of these children and their families to improve their quality of life.

The purpose of this retrospective single-center study was to determine the prevalence and reasons for visiting the ED, the management, outcome, and quality of care received in a Tertiary Care Pediatric Hospital, where the PPC facility is strongly integrated and cooperating with the ED.

**Methods:**

We collected data on the 775 pediatric ED visits performed in Padua Pediatric ED between 2006 and 2023 by 85 children under the care of the regional center for PPC in Veneto, Italy.

**Results:**

Median number of visits per patient was 2.0 per year, 33.4% resulting in hospital admissions. Most frequent reasons for ED visits were respiratory conditions (28.9%), followed by medical device malfunctions (18.3%). Other data included PPC specialist involvement, ED referral, time of arrival, color code, and type of assistance required. Over the years, there has been an increased number of ED visits, admissions, medical device malfunctions, hours spent in the ED, and involvement of PPC specialists. No significant influences were noted during the COVID-19 pandemic period.

**Conclusion:**

These data highlight potential areas of intervention to enhance emergency care management for CMC, such as early PPC specialist involvement with the home care network activation, a specific training of ED providers, and a dedicated service for the management of device malfunctions.

Children with medical complexity (CMC) represent an increasing pediatric population characterized by multiple medical conditions, a diverse spectrum of needs, and frequent medical device utilization.^1^ A wide range of pathologies may be implicated and these children constantly require specialized services that support basic life functions, round-the-clock assistance from parents/caregivers, and, in most cases, reliance on medical technology replacing a vital body function.^2^ Children with medical complexity (CMC) visit the emergency department (ED) frequently.^3–5^ Studies from the United States and Canada show that CMC accounts for about 20% of all ED visits and an increasing share of hospitalizations and resource use.^3–7^ Limited European data exists regarding children with medical complexity (CMC) in the ED, with one Italian study highlighting their higher clinical severity, more interventions, and increased hospitalization rates compared with general pediatric patients.^8^ Managing CMC in the ED is challenging due to their medical fragility, providers’ inadequate training, time constraints, and insufficient equipment.^3–10^ ED staff often struggle with understanding complex medical histories, coordinating care, and accessing vital patient information.^9–11^

Pediatric palliative care (PPC) aims to address the needs of these children and families and to improve their quality of life by establishing a solid network of assistance and care. A recent report indicates that 20,540 to 32,864 children in Italy require PPC, accounting for 34 to 54 children/100,000 inhabitants, of whom 18/100,000 require a specialized PPC service.^2,12^

Our institution, the University Hospital of Padova, Italy, serves as the Veneto Region’s Reference Center for Pediatric Palliative Care (PPC). We provide 24/7 multidisciplinary support, with over 90% of care delivered at home, coordinated with local services. For needs not met by telemedicine, families can access our Pediatric Hospice or the Pediatric Emergency Department, ensuring continuous, relationship-centered care.

Overall, there is a lack of comprehensive studies investigating the specific challenges and outcomes of CMC patients in an ED setting, empowered with an active home care network and PPC service. Our study, the VADEMECUM Project, aimed to determine the prevalence and reasons for CMC visiting the ED, their management, outcomes, and the type of care received in a Tertiary Care Referral Pediatric Hospital, where the PPC facility is strongly integrated with and collaborates with the ED.

Secondary outcomes were the description of changes over the years and during the COVID-19 pandemic, as well as the economic burden. These data were analyzed to identify possible strategies to reduce time spent in the ED or to prevent ED visits in the future.

## METHODS

We carried out a retrospective single-center study on selected CMC in a tertiary care referral pediatric hospital. The study population included patients (from 0 to 21 y old) followed by the hospital PPC service (which is the regional PPC and Pain Service center in the Veneto region, Italy), who visited the pediatric ED of Padua University Hospital in the time period between January 1, 2006 and December 31, 2023. Of the whole group of patients followed by our PPC service, we included in this study only CMC who were residents in the province of Padua, for whom the pediatric ED represented the nearest access point for emergencies. Our pediatric ED has an annual census of ∼25.000 patients per year. We excluded patients who are referred to our specialist pediatric ED, but who do not reside in the close referral area. The study was carried out in accordance with the institutional review board’s ethical standards and the Helsinki Declaration. The study protocol has been approved by the Institutional Review Board of the Azienda Ospedaliera Università di Padova (375n/AO/23).

For each patient, the electronic medical records of every ED visit during the study period were initially reviewed manually by the research team. Subsequently, a GPT-based model (GPT-4o), accessed through the OpenAI API in R system language, was utilized to assist in the secondary review of data extraction, thereby enhancing its accuracy by identifying and comparing potential inconsistencies between the data extracted by the human operator and those reported by the GPT-based model.

Data included number of ED visits per year, reason for ED access, decision to refer to the ED (caregiver initiative/medical referral), ED arrival time (day/night and weekend/weekdays), time spent in the ED, outcome (discharge, hospital admission), vital signs, medications administered, diagnostic tests performed, and final diagnoses.

The number of CMC ED visits per year and their outcomes were standardized for the total number of ED visits per year in our ED.

Involvement of known provider (PPC specialist on call) was also recorded, as well as color code at triage and discharge/admission, ranging from most critical (with highest priority to be seen by a physician) to least, in the following order: red, orange, yellow, green, white (in addition to blue, representing a particular set of programmed nonurgent visit).

We also collected data regarding the economic cost of each ED visit, based on the estimation of cost reported in each medical record at the moment of the patient’s discharge (usually fully covered by the institution, without any economic contribution required from the patient).

### Statistical Analysis

Descriptive statistics were reported as median and interquartile range (IQR) for continuous variables, and absolute numbers and percentages for categorical data. The association between color code at triage and hospitalization as an outcome of the ED visit was analyzed using univariate logistic regression, with odds ratios (OR) and their 95% confidence intervals (95% CI). In the regression analysis, yellow and orange codes were combined into a single group since the orange code was introduced later, after 2017. Statistical significance was defined as *P*<0.05.

Furthermore, temporal trends of these data were reported to highlight variations linked to different time periods (eg, pandemic period due to COVID-19).

All statistical analyses were performed using the R System version 4.3.3.

## RESULTS

The study sample included 85 CMC, who had as their primary referral center the pediatric ED of Padua University Hospital. We collected a total of 775 ED visits over the study period. The median number of ED visits per year for CMC (standardized for the total number of pediatric ED visits per year in our center) was 2.0 (mean: 2.34), which accounts for up to 1% of total ED visits per year at our center.

Median number of ED visits per year from 2006 to 2023 in the pediatric ED of Padua was 24.200/year (including the deflection of ED visits due to the pandemic period and the following increase with the maximum number of 28,416 ED visits reached in 2023).

**FIGURE 1:**
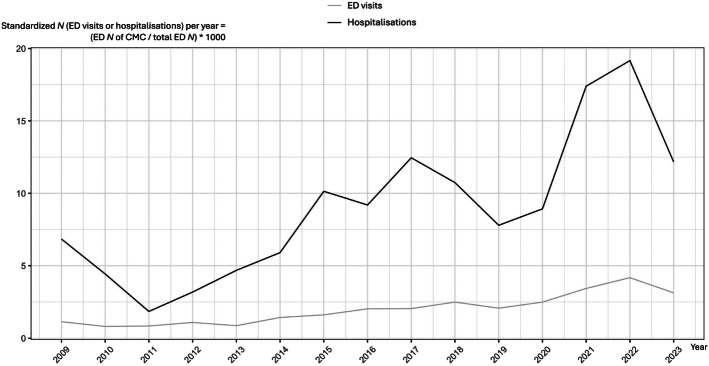
Trend of ED visits and hospitalization per year, standardized.

While 18.8% of CMC never visited the ED during the study period (16/85), an equal number (18.8%, 16/85) accessed the ED more than 5 times per year. Over the years, there was an increased number of ED visits for CMC as well as an increased number of hospital admissions following ED visits (Fig. 1).

### Outcome (Discharge or Hospital Admission)

A total of 33.4% ED visits resulted in hospital admission (259/775), while 65.8% of patients were discharged (510/775). A small number (0.8%, 6/775) was not classified either in discharge or admission due to refusal of admission by the caregivers or departure from the ED without waiting for medical directives.

### Reason to Visit the ED

The most frequent reasons for ED visits were represented by respiratory conditions (28.9%), followed by medical device malfunction (18.3%). All reported reasons to visit the ED are summarized in Table 1.

**TABLE 1:** Reason to Visit ED

	Overall 775 ED Visits	Pre-COVID 461 ED Visits	COVID 132 ED Visits	Post-COVID 182 ED Visits
Reason to Visit ED	n (%) of ED Visits	n (%) of ED Visits	n (%) of ED Visits	n (%) of ED Visits
Respiratory	224 (28.9)	138 (29.9)	36 (27.3)	50 (27.5)
Medical device malfunction	142 (18.3)	39 (8.5)	34 (25.7)	69 (37.9)
Neurological	101 (13)	72 (15.6)	19 (14.4)	10 (5.5)
Infectious (nonrespiratory)	78 (10.1)	51 (11.1)	15 (11.4)	12 (6.6)
Gastroenterological	74 (9.5)	52 (11.3)	8 (6.1)	14 (7.7)
Miscellaneous (*dental, dermatological, genitourinary, metabolic, nontraumatic osteoarticular, or vascular*)	59 (7.6)	37 (8.0)	8 (6.1)	14 (7.7)
Trauma	50 (6.5)	40 (8.7)	5 (3.8)	5 (2.7)
Programmed visit	20 (2.6)	138 (29.9)	0 (0.0)	0 (0.0)
Pain	20 (2.6)	6 (1.3)	6 (4.5)	8 (4.4)
Cardiological	7 (0.9)	6 (1.3)	1 (0.8)	0 (0.0)

Overall: January 30, 2006 to October 12, 2023 (775 ED visits).

Pre-COVID: January 30, 2006 to February 23, 2020 (461 ED visits).

COVID: June 4, 2020 to March 29, 2022 (132 ED visits).

Post-COVID: September 4, 2022 to October 12, 2023 (182 ED visits).

ED indicates emergency department.

Respiratory problems were also the most frequent condition leading to hospital admission (44% of total admissions, 114/259), followed by neurological conditions (22%, 58/259), while medical device malfunction was the most common reason to visits the ED for patients that were eventually directly discharged from the ED (26%, 133/516). In addition, patients with frequent device malfunctions were also those with a higher number of ED visits per year.

### Time of Arrival

Time of arrival was differentiated in daytime (7 AM to 7 PM; 73%, 566/775) or nighttime (7 pM to 7 AM; 27%, 209/775) and in weekdays (monday to friday; 76% 592/775) or weekends/other festivities such as Christmas, national festivities, etc. (24%, 183/775). Overall, approximately one fourth of CMC presented during night time or during weekends.

### Triage Code

**FIGURE 2:**
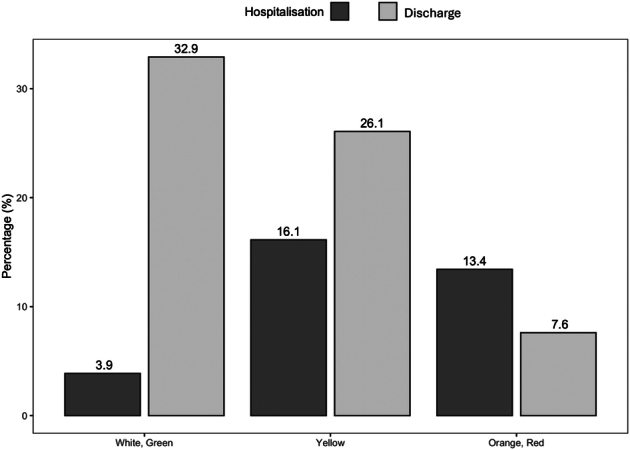
Admission per color code at triage.

Higher priority triage color codes, such as orange or red, were more likely to result in hospital admission [code red: OR: 27.97 (*P*<0.01, 95% CI: 12.63-66.97); code orange/yellow: OR: 5.66 (*P*<0.01, 95% CI: 3.13-11.17); code green: OR: 0.89 (*P*=0.76, 95% CI: 0.42-1.98)] (Fig. 2).

At triage, the most frequently attributed color code was yellow (42.2%, 327/775), as seen in Table 2, where there are also reported the percentages of each color code at the moment of discharged/hospital admission (this code could have been modified or confirmed at the end of each ED visits, if the physician who took care of the patient did not consider the original color code representative of child’s actual clinical condition).

**TABLE 2:** Color Code

Color Code	In Triage, n (%) of ED Visits	At Outcome, n (%) of ED Visits
White	56 (7.2)	107 (13.8)
Green	178 (23.0)	150 (19.4)
Yellow	327 (42.2)	317 (40.9)
Orange	95 (12.3)	95 (12.3)
Red	68 (8.8)	69 (8.9)
Blue	51 (6.6)	31 (4.0)
NA	0 (0.0)	6 (0.8)

ED indicates emergency department; NA, not applicable.

### ED Referral

In most cases self-presentation to the ED was reported (73.8%, 572/775), while in 10.7% of cases (83/775) the referral was made by the PPC specialist, in 6.2% (48/775) by the pediatric primary care practitioner and in 9.3% (72/775) by other physicians or from other hospitals.

### Time Spent in the ED

Time spent in the ED progressively increased over the years, with a median time of 4.0 hours (range: 1 to 31 h). Following stratification based on outcome, the median time spent in the ED resulted longer (7.0 h) when the outcome was hospital admission.

### Type of Assistance Provided

The type of assistance received in the ED was differentiated according to diagnostic tests, such as laboratory tests (blood, urine, liquor, microbiological samples), radiological tests (x-ray, CT scan, MRI, echographies), other tests (electrocardiogram, echocardiography, electroencephalogram), specialist consult (surgical, cardiological, neurological, ENT, etc.), oxygen therapy or ventilation, medications/fluids administration for the treatment of the acute condition, nasogastric tube replacement and others.

The most frequently performed investigations were laboratory tests in 54.2% of ED visits, and specialist consults (41.0%). The most frequent therapeutic intervention was medication/fluids administration (43.9%). The complete list is reported in Table 3.

**TABLE 3:** Type of Assistance Received

Type of Assistance	n (%) of ED Visits
Lab tests	420 (54.2)
Medications/fluids	340 (43.9)
Specialist consult	318 (41.0)
Radiological tests	291 (37.6)
Oxygen/ventilation	76 (9.8)
None	48 (6.2)
Other tests	38 (4.9)
Nasogastric tube replacement	33 (4.3)
Immunization shots in a protected environment	20 (2.6)
Other	3 (0.4)

ED indicates emergency department.

Most patients received at least one procedure or test during their time in the ED, mostly lab tests or radiological tests (Table 4).

**TABLE 4:** Number of Procedures/Tests Per Visit

Procedure/Tests Per Visit	n (%) of ED Visits
0	48 (6.2)
1	284 (6.7)
2	199 (25.7)
3	119 (15.4)
4	125 (16.1)

ED indicates emergency department.

### PPC Specialist Involvement

**FIGURE 3:**
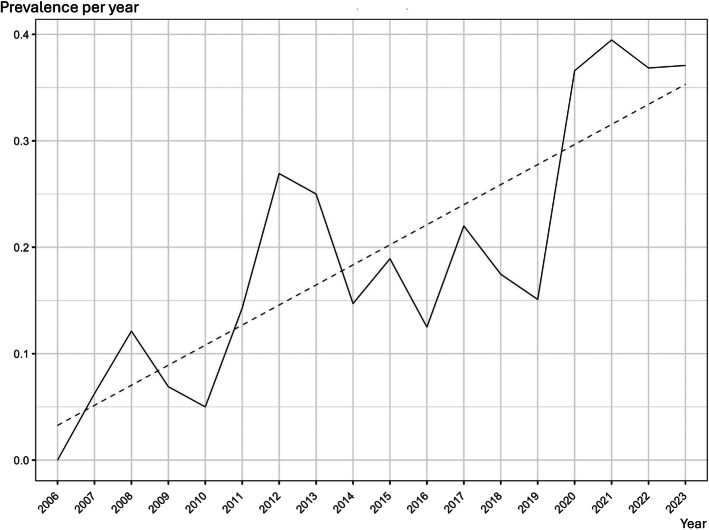
Trend of prevalence of PPC specialist involvement during ED management.

The involvement of the PPC specialist was required by ED physicians in 24.6% of ED visits (191/775), progressively increasing over the years, from 8.9% of visits (11/124) in the time period 2006-2011 and reaching 31.9% (139/436) in 2018-2023 (Fig. 3).

### Temporal Trend

Over the years, we recorded an increase in the number of ED visits, hospital admissions, ED visits due to device malfunctions, time spent in the ED, and increased involvement of PPC specialists. Temporal trend analysis focused on the COVID-19 pandemic period (March 11, 2020 to March 31, 2021) showed no differences in the number of ED visits or in the reasons to require ED care. As an example, respiratory conditions as a reason to visit ED accounted for 30% (38/461) of ED visits pre-COVID, 27% (36/132) of ED visits during COVID, and 27% (50/182) of ED visits post-COVID.

### Economic Burden

The economic cost for our institution of each ED visit was not always reported; therefore, the following data are likely underestimated. According to available data for 2023, the total annual cost for 52 ED visits of medically complex children in our pediatric emergency department was 4402€. Thirty-four of these visits were due to medical device complications for a total of 1989€. Regarding costs linked to medical device malfunctions, the most frequently reported cases required a surgical/specialist consult in addition to usual standard ED care, with a resulting cost of 45.5€ per visit. If additional investigation or procedures were performed, the cost per visit could reach up to 277€. If we applied the most reported cost to the 142 visits due to medical device malfunctions performed over the years 2006-2023, the total amount of expenses can be estimated to have been at least 6461€ (358.9€/year).

## DISCUSSION

This is one of the first European studies describing the trend and prevalence of CMC ED visits over the last 18 years in a Pediatric Tertiary Care Center.

### Comparison to Available Literature

We reported an increased number of CMC ED visits per year, as well as hospital admissions, although CMC ED visits account for only 1/1000 of the total ED visits per year in our institution. We elected to exclude patients transferred from other hospitals to focus in the geographical area under the direct care our PPC service and ED, so the total number of CMC patients assessed in our ED is most probably higher; however, it unlikely would reach the percentage reported in a north American retrospective study where CMC visits at the ED were estimated to be up to 20% of the total visits.^4,5,13^ The growing number of visits over the years is probably due to the progressive increase in CMC population and care complexity.^14^ In our center, a steadily increasing trend was observed over time in CMC referrals to our PPC service, with 109 new patients referred only in the year 2022 compared with the 37 patients referred in 2008 (195% increase), as reported in Schiavon et al.^14^

Similar to previous literature^4–6^ and national Italian data,^9^ the most common reason for ED visit in our center was the presence of respiratory symptoms (28.9%), followed by malfunction of a medical device (18%). Redirect device malfunctions to a separate, more specialized setting (eg, experienced staff, specialist consult, and proper equipment availability) such as our PPC Center and/or empowering home care assistance, may offer higher quality of care, reduce the exposure for these fragile patients to a high risk setting such as the ED and contribute to decrease the economic burden of their care in the ED. The possibility of intervening by reducing the burden of this large percentage of ED visits, therefore, remains an important point of improvement to be taken into consideration for home care services.

Respiratory symptoms are both the main reason leading to an ED visit and the most frequent cause of hospital admission. A total of 33.4% ED visits ended up in hospital admission compared with 45.7% of the national cohort reported.^14^ These data are similar to other reports and suggest that more than 1 in 4 children with CMC who visited a children’s hospital ED experienced hospital admission.^4–6^ A systematic review in 2021 highlighted how CMC presented indeed higher rates of hospital admission, as well as ED revisit within 72 hours and adverse drug events.^15^

In our institution, 62.9% of the visits (compared with the national report of 51.4%) were classified as “urgent,” with a red/yellow triage code.^9^ These data suggest that CMC ED visits are indeed due to urgent nondeferrable reasons. The number and reasons for visiting the ED have also remained similar during the pandemic, suggesting that most ED visits for CMC were necessary and could not be deferred. Furthermore, in 20% of cases, it was the same specialist doctors or primary care pediatricians who sent the patient to the ED, underlining the continuous contact with the patient at home before entering the hospital. It is also important to underline that 20% of the CMC taken care of by the PPC service have never made any access to the ED, while a similar percentage presented to the ED more than 5 times per year.

Time spent in the ED is also gradually increasing; we hypothesize this is most likely due to the fact that ED settings are frequently insufficient for effectively managing CMC acute and chronic demands due to a lack of equipment, proper settings, and staff. Unfortunately, in the literature, there are few data on this topic, so it is difficult to compare our trend with other centers. More time spent in the ED means longer exposure to infectious risks for these vulnerable patients, an increased burden of care for their caregivers, and a greater budgetary burden on the health care system. Little is known about the potential to influence treatment, caregiver self-efficacy, and community hospitalization risk following an ED visit. Based on the existing literature, the most effective methods for minimizing ED visits and resource consumption were 24-hour access to a known clinician, like a PPC specialist, and next-day appointments, outpatient interventions, and home care assistance.^15^ The most consistent elements for successful intervention involved continuous (24 h a day/7 d a week) access to health care providers which is a facility that in our center is already present, but we are considering to implement expedited access to a dedicated ambulatory appointment for nonurgent codes (which approximately account for 20% of ED visits in our center, if we exclude the 62.9% which are nondeferrable and the 20% related to medical devices disfunction that could be potentially taken to another dedicated ambulatory or home service network).

Data regarding the involvement of the PPC specialist (both for the ED referral and the management in the ED) may be underestimated since it is not always explicitly reported in the ED reports; however, it is increasing over the years on account of the 24/7 availability for a consult of the Regional Center of PPC and Pain Service in Veneto (both for other physicians and both directly for patients’ families). Focusing on preventing ED visits should be a priority; however, when acute care is required, ED physicians should be able to rely on an evidence-based approach and the support of an integrated and reliable PPC network to provide high-quality care. Most patients followed by our PPC center already have an advanced care plan when they arrive in the ED or if they have to use the emergency service via ambulance. All the advance care plans are shared with the territorial retrieval service, and the on-call PPC specialist is 24/7 available to discuss with them the attitude and clinical management if needed.

### Limitations

Data are limited to a single academic tertiary care children’s hospital. Since our pediatric ED represents a separate department in the pediatric hospital, children are taken care of entirely by experienced pediatricians, pediatric emergency physicians, and pediatric nurses. Different subspecialty consultations are almost constantly available, and 24-hour access to a PPC specialist and a known provider is easily acquirable. The availability of resources and highly trained providers significantly influences the challenges faced and the solutions' applicability, which may differ in other, less specialized settings. However, this offers the possibility to highlight which interventions could be effective for improving the quality of received care and reducing overall ED visits. Another potential issue is that the color code assignment during triage may be based on the triage provider. It is reported that for this kind of patient, there is a natural tendency to overtriage.^14^ Therefore, the triage code may not be representative of the actual severity of the condition, but rather of the priority of these particular patients for attention in the ED flow. As an example in our study, for the same child with multiple visits for the same issue (medical device malfunction), each time with similar vital signs and no different additional comorbidities, the assigned color code was highly variable. While some triage providers assigned a priority based on the baseline fragile condition of the patient, others have preferred to base the color code on the current acute condition that has led to the ED visit. To minimize this bias, this is why we reported the triage on ED presentation and on discharge.

### Future Perspectives

The implementation of a dedicated code or care pathway may offer a more appropriate recognition and priority assessment for CMC in triage. In addition, specific training for ED health care providers may help them identify acute and priority conditions in CMC and standardize their management, as already reported in different centers.^5,11,13^ Lastly, despite having limited available data on ED visits economic costs, it is possible to establish that a large amount of these expenses is due to the management of medical device complications. These findings suggest how the improvement of device malfunction management may be the most effective in reducing the costs of care.

These results are consistent with previous published literature that highlights the need for trustworthy medical information on CMCs during medical emergencies, as reported by the review of Pulcini et al,^12^ who reported that parents/caregivers of CMC believed that insufficient data exchange occurred between parents and physicians during medical emergencies. Pediatric emergency medicine doctors observed similar issues, and several advised that sending treatment advice electronically (rather than physically searching the electronic health record) could improve emergency care. Building on a strong complex care program, and maybe implementing telemedicine solutions as indicated by Mosquera et al^16^ and Lin et al^17^ can empower the home visiting program, ultimately reducing ED visits. Furthermore, dedicated patient-oriented objectives like feasibility, acceptability, and user-centered design strategies for outpatient therapies for CMC in an emergency situation require additional research.

## CONCLUSIONS

CMC are a growing population and so are the challenges of their appropriate care in the emergency department. We believe that the involvement of the PPC specialist is crucial in supporting the ED physician in the management of these patients in their acute care, as well as directly supporting the family with a 24/7 phone call availability to provide medical directives and potentially prevent those ED visits that may be avoided, even through the provision of home visits.

Training of ED providers, an appropriate priority assessment tool in triage, and specialized dedicated service (ambulatory or home care) for the management of medical devices may improve the quality of provided care and reduce the financial cost of CMC management in the ED.

These data need should be further supported by additional studies with comparison to the general population, since this descriptive study included only CMC already enrolled in our PPC service; therefore, it does not report any data on what is occurring with those who are not enrolled.
